# Multidisciplinary rehabilitation and eye tracker-assisted communication in locked-in syndrome: a case report and literature review

**DOI:** 10.3389/fnhum.2026.1844573

**Published:** 2026-08-12

**Authors:** Qi Zhang, Weibo Tian, Xinyu Miao, Kaidan Chi, Zhe Chen, Xinyi Li, Wei Liu, Zhongliang Liu, Xiaoqin Duan

**Affiliations:** 1 Department of Rehabilitation Medicine, The Second Hospital of Jilin University, Changchun, Jilin, China; 2 Department of Neurology and Neuroscience Center, The First Hospital of Jilin University, Changchun, Jilin, China; 3 Department of Obstetrics and Gynecology, The First Hospital of Jilin University, Changchun, Jilin, China; 4 Cancer Center, The First Hospital of Jilin University, Changchun, Jilin, China; 5 Department of Thoracic Surgery, The First Hospital of Jilin University, Changchun, Jilin, China

**Keywords:** case report, communicative impairment, eye tracker, locked-in syndrome, multidisciplinary rehabilitation

## Abstract

**Background:**

Locked-in syndrome (LIS) is a rare and severe neurological condition characterized by quadriplegia and anarthria with preserved consciousness. Communication impairment is a major barrier to psychosocial adaptation and long-term rehabilitation. A new rehabilitation strategy is needed to restore multi-sensory impairments.

**Case presentation:**

A patient underwent eye tracker therapy as part of a comprehensive multidisciplinary rehabilitation program following the diagnosis of LIS, which aimed to improve ocular scanning and gaze patterns to enable the patient to communicate using ocular motor signals. The effects and applications of eye-tracking in the neurorehabilitation of patients with Locked-In Syndrome (LIS) were synthesized in a brief review. After a three-month period of rehabilitation, the patient showed improved communication ability and better psychosocial adaptation, suggesting that eye-tracker assisted communication may be a useful adjunct within multidisciplinary rehabilitation for selected patients with LIS.

**Conclusion:**

This case highlighted the importance of eye trackers and multidisciplinary rehabilitation for LIS to address communication disorders and other dysfunctions in patients, which would help patients return to their families and society as soon as possible.

## 1 Introduction

Locked-in syndrome (LIS) is one of the most severe and rare chronic neurological conditions, which was first described in Plum and Posner (1972). According to the preservation of ocular and other voluntary movements, LIS can be classified into classic, incomplete, and complete forms (Hocker and Wijdicks, 2015). Classic LIS patients are almost completely immobile, with preserved consciousness and cognition, and vertical eye movements or blinking may serve as the only available channels for communication (Bauer et al., 1979). Incomplete LIS refers to cases in which additional voluntary movements are preserved beyond vertical eye movements or blinking. Ischemic infarction or hemorrhage in the vertebrobasilar arterial territory is the main cause of LIS (Patterson and Grabois, 1986), but lesions may also extend beyond the brainstem and involve supratentorial structures, white matter fiber tracts, or cortical regions (Horan et al., 2019; Leonard et al., 2019; Sarà et al., 2018). This dissociation between preserved awareness and severely impaired motor expression makes LIS a unique and challenging condition in neurological rehabilitation.

Patients with LIS are associated with increased mortality, poor functional prognosis, long-term dependence, and substantial family and socioeconomic burden (Hocker and Wijdicks, 2015; Patterson and Grabois, 1986; Smith and Delargy, 2005). Despite extensive investigation, the prevalence and pathogenesis of LIS remain incompletely understood. Apart from the management of acute lesions and the prevention of secondary complications, no curative treatment is currently available for established LIS (Schnetzer et al., 2023). Bauer et al. suggested that achieving complete functional recovery in classic LIS cases is nearly impossible, and treatment is often limited to symptom improvement and maintenance of vital functions (Bauer et al., 1979). Therefore, long-term management of LIS should extend beyond survival and physical stabilization to include rehabilitation, nursing care, and psychological support. Although LIS causes profound physical disability and dependence, quality of life should not be inferred solely from motor impairment. Previous studies have shown that many patients with LIS may report an acceptable quality of life when effective communication, social support, psychological adaptation, and appropriate care are available (Lulé et al., 2009; Kuzma-Kozakiewicz et al., 2019). Because patients with LIS often retain awareness and cognitive function but lose the ability to speak or move, communication impairment represents a central barrier to expressing needs and emotions. Improving communication may be an important and feasible strategy to support the quality of life in patients with LIS.

Accordingly, restoring reliable communication is a central goal of multidisciplinary rehabilitation for patients with LIS. Conventional approaches, such as blinking-based responses and alphabet boards, can provide basic communication, but they are often slow, fatiguing, and highly dependent on caregiver interpretation. Recently, assistive communication technologies have been increasingly explored, mainly including electroencephalography-based systems, electrooculography-based interfaces, brain-computer interfaces, and eye-tracking devices. For patients with preserved voluntary eye movements, eye-tracking technology is particularly relevant clinically. It directly uses the residual ocular motor pathway, is non-invasive and relatively portable, and can convert gaze fixation into computer-based language output. Therefore, eye-tracker-assisted communication may provide an alternative communication strategy for selected patients with LIS within integrated post-stroke rehabilitation and long-term care.

Here, we report a patient with LIS secondary to vertebrobasilar ischemic stroke who presented with quadriplegia and severe communication impairment. The patient underwent a multidisciplinary rehabilitation program in which eye-tracker-assisted communication was introduced as the primary augmentative and alternative communication method. This case highlights the potential value of integrating eye-tracking technology into comprehensive rehabilitation for selected post-stroke patients with LIS. By combining a detailed case presentation with a brief review of eye-tracker-assisted communication in locked-in states, this report aims to provide practical experience for improving communication ability, psychosocial adaptation, and long-term rehabilitation outcomes in patients with LIS.

## 2 Case presentation

### 2.1 Clinical history and diagnostic assessment

**Figure 1:**
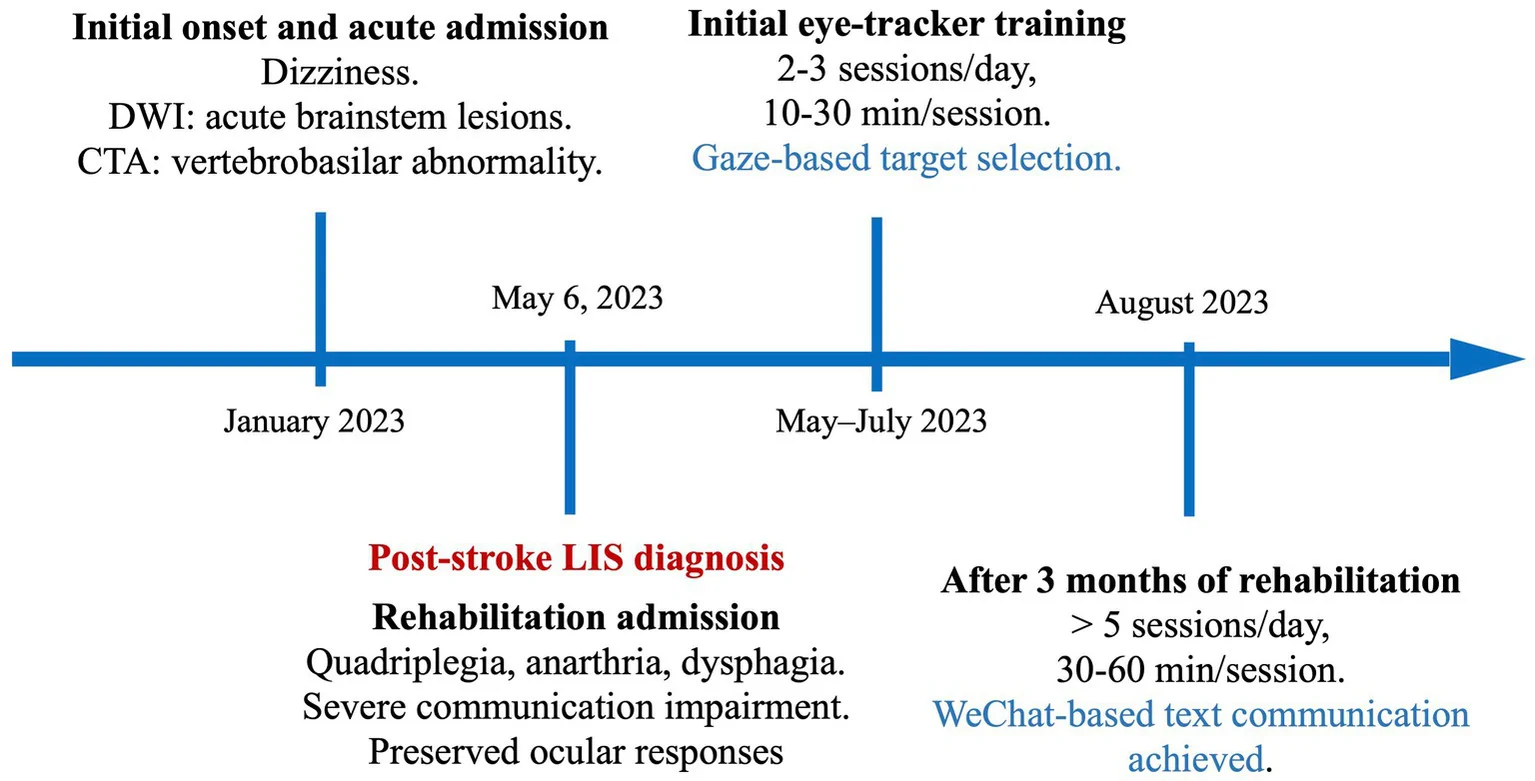
Timeline of clinical course and eye-tracker-assisted rehabilitation in post-stroke locked-in syndrome.

**Figure 2:**
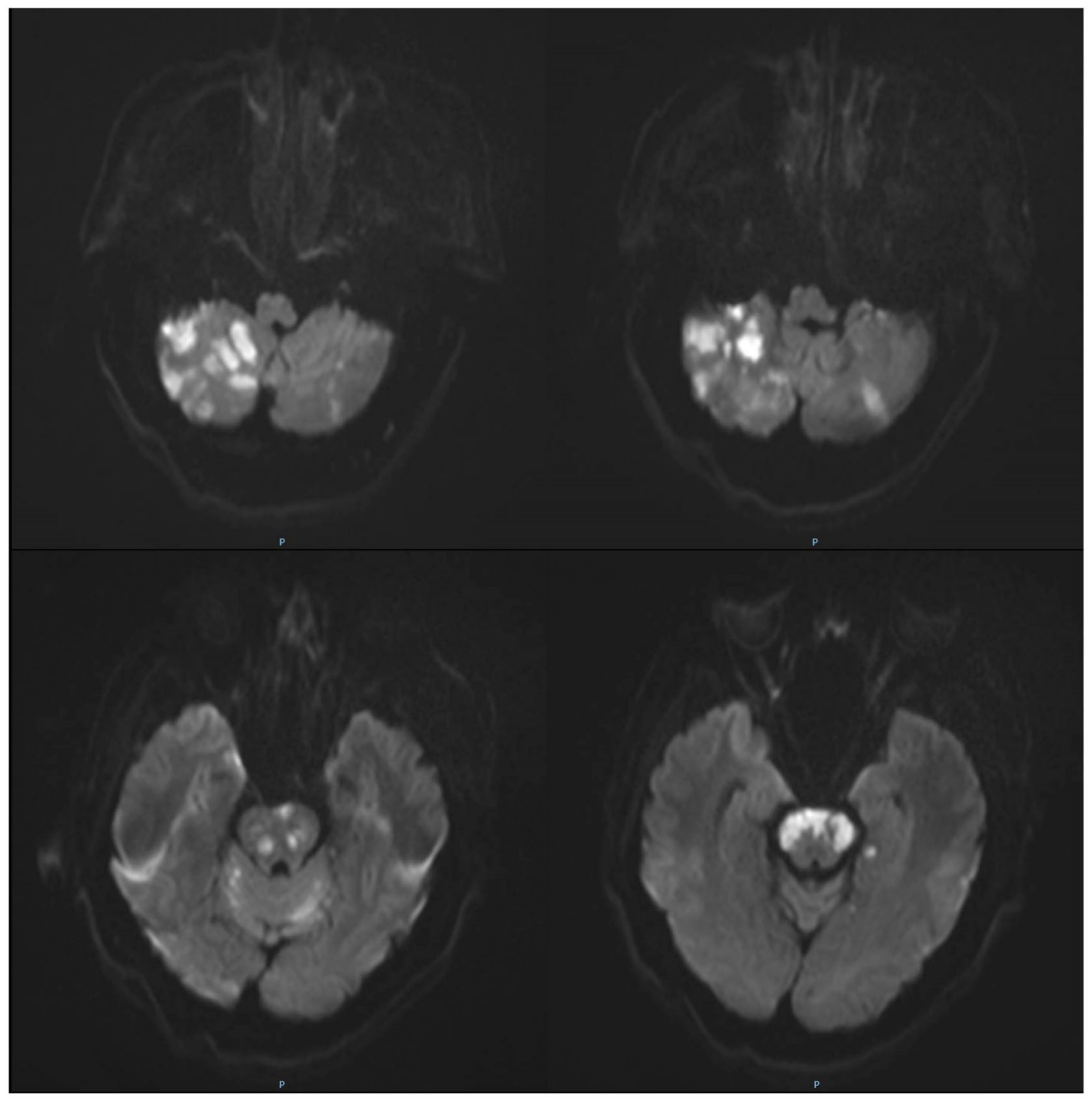
The diffusion weighted imaging (DWI) of the head on January 10, 2023. Brainstem, bilateral cerebellar hemispheres, left hippocampal region, the periventricular region adjacent to the posterior horn of the left lateral ventricle, and right thalamus showed punctate hyperintense lesions.

**Figure 3:**
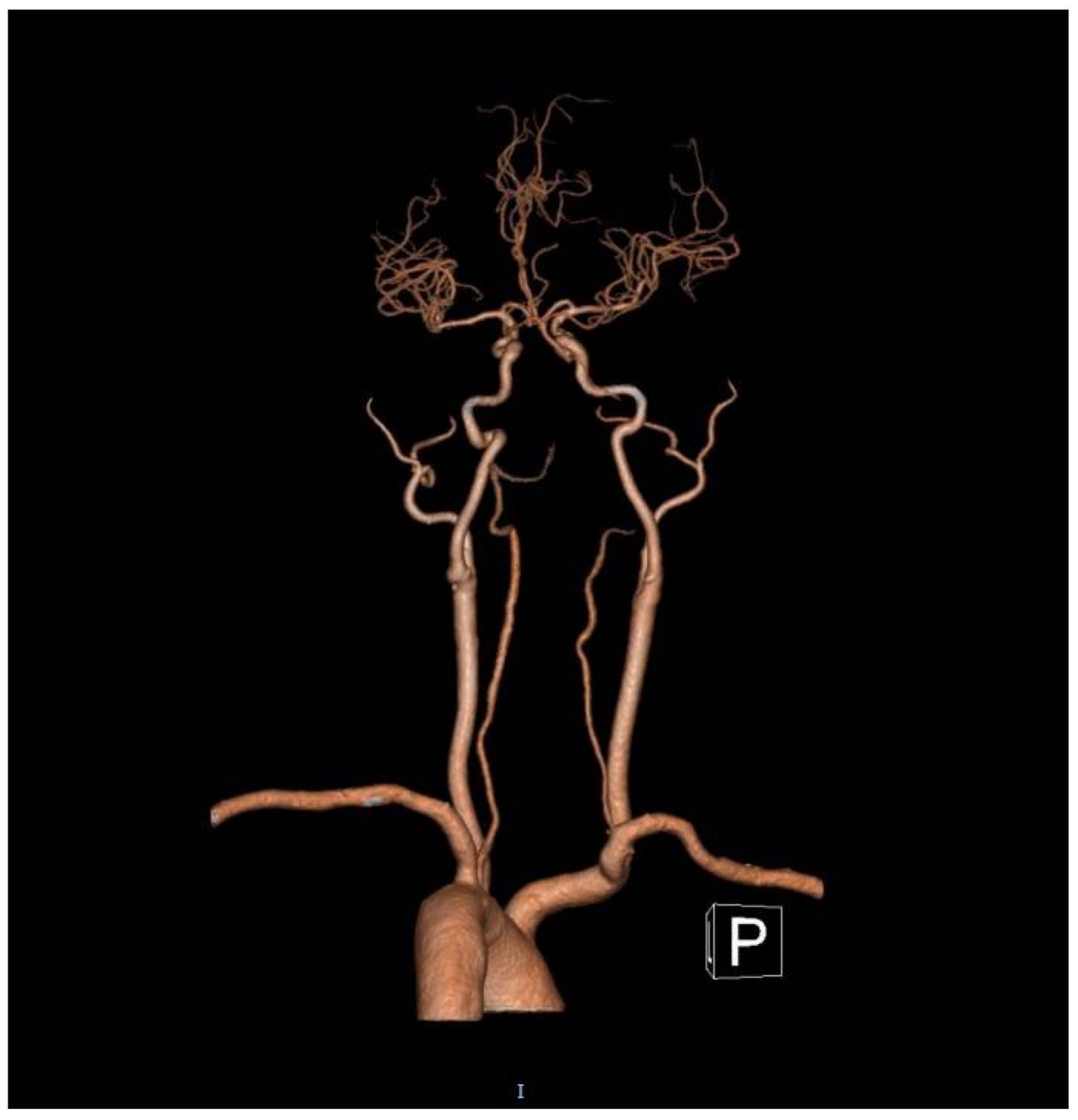
The computed tomography angiography (CTA) of the head on January 12, 2023. Both sites of bilateral vertebrobasilar intersection were not visible.

The overall clinical course and eye-tracker-assisted rehabilitation timeline are summarized in Figure 1. A 59-year-old man was admitted to the Department of Rehabilitation Medicine at the Second Hospital of Jilin University on May 6, 2023, presenting with symptoms of sudden quadriplegia and dysarthria without apparent causes over 4 months. Actually, he sought treatment at the China-Japan Union Hospital of Jilin University 4 months ago for severe dizziness, inability to stand, and profuse sweating. MRI diffusion-weighted imaging (DWI) revealed punctate high-intensity signals with indistinct boundaries in the brainstem and other anatomical regions (Figure 2). Computed tomography angiography (CTA) of the head showed that both sites of the bilateral vertebrobasilar intersection were not visible. A plaque formation was observed in the initial part of the right vertebral artery wall, where the lumen was severely narrowed (Figure 3). However, the patient’s condition worsened 7 days later, and the patient was transferred to the ICU and underwent a tracheotomy.

After systematic treatment, his condition improved, but still with communication impairment and the use of the gastric tube and tracheostomy tube. When he presented to our hospital in May 2023, he had quadriplegia, anarthria, dysphagia, poor nutritional status, and severe communication impairment. There was a six-year history of hypertension with a highest value of 170/100 mmHg, which was 155/85 mmHg after intermittent use of amlodipine besylate tablets. Thereby, we conducted comprehensive rehabilitative assessments based on the International Classification of Functioning, Disability and Health (ICF) theory. Neurological examination showed almost complete paralysis, with preserved spontaneous eye opening and residual ocular motor responses. Although verbal and limb motor responses were absent or markedly limited, repeated clinical assessment suggested preserved consciousness and awareness. The bilateral biceps reflex was asymmetric, and the Babinski sign, Kernig sign, and Brudzinski sign were all positive. The Modified Ashworth Scale indicated a muscle tone of level 2 in both upper and lower extremities, with no significant restriction in passive joint range of motion. Moreover, he was unable to walk, and his sitting and standing balance could not be maintained. Besides, the ability of the daily life (ADL) score was 0, indicating complete dependence on daily life. According to the diagnostic criteria established by the American Congress of Rehabilitation Medicine (1995), the patient was diagnosed with locked-in syndrome, cerebral artery stenosis, cerebral infarction, absolute dependence of ADL, grade 2 hypertension, dystonia of the extremities, and dysphagia. The initial rehabilitation goal was set to improve the patient’s communication ability.

### 2.2 Eye-tracker-assisted multidisciplinary rehabilitation and outcomes

**Figure 4:**
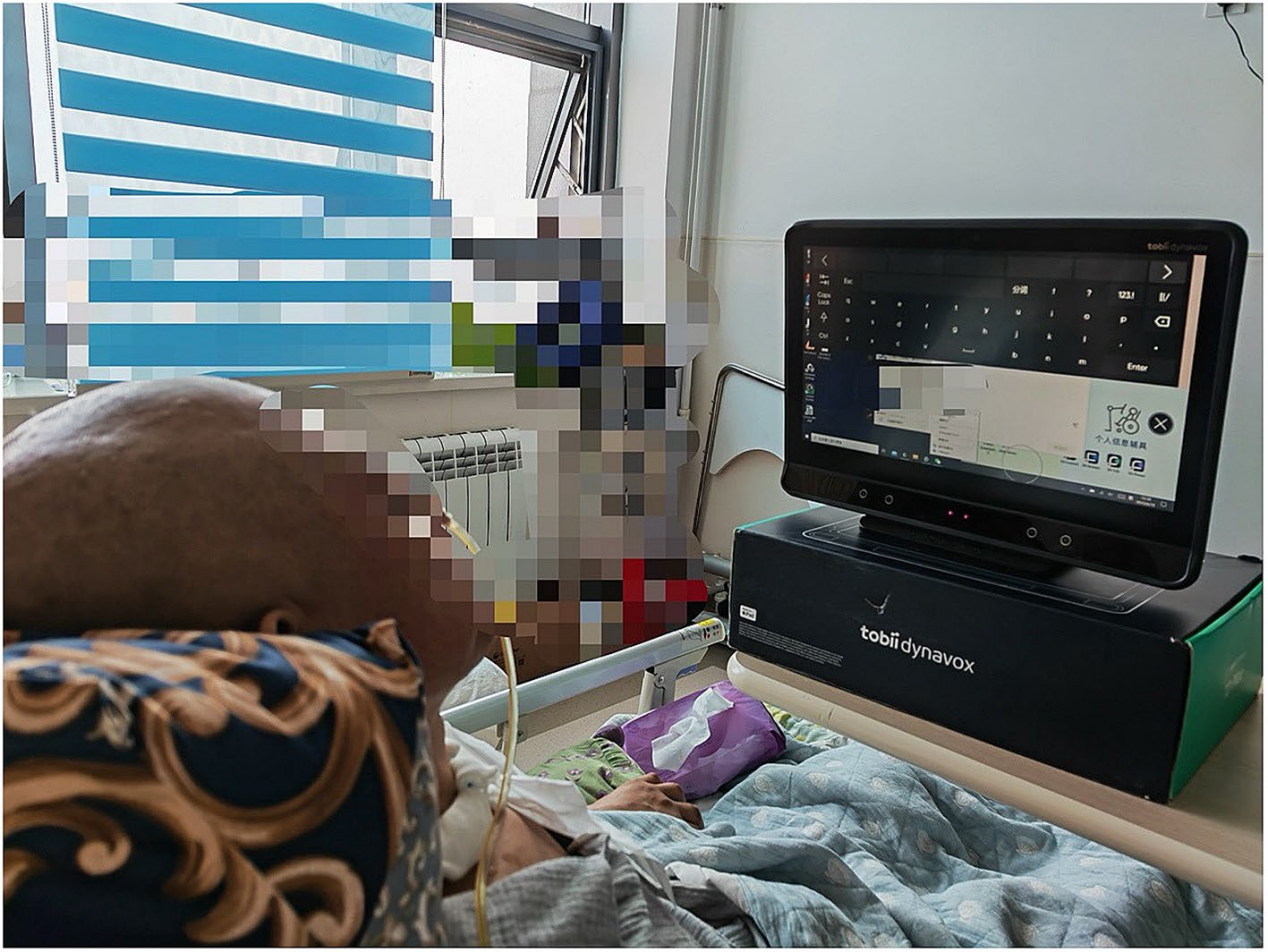
Eye tracker-assisted communication training in the patient with locked-in syndrome. The patient participated in bedside eye tracker-assisted communication training using a Tobii Dynavox device as part of the multidisciplinary rehabilitation program. The system enabled gaze-based target selection and communication, providing an alternative means of interaction for the patient. The image has been anonymized to protect patient privacy.

The training involved the use of Tobii PCEye, the latest generation of eye-tracker communication AIDS, and communication board modules of Tobii PCEye (Tobii Dynavox PCEye 5, Tobii Technology AB, TobiiDynavoxLLC, Pittsburgh, U.S.). The device was connected to a 15-inch Windows laptop, and eye movements were tracked using a computerized infrared eye tracker, sampling at 60 Hz, which converted the movements into computer signals. The patient was positioned 70 cm from the monitor with a 30° visual angle (Table 1; Figure 4).

**Table 1:** Eye-tracker training parameters and outcomes.

Stage	Training parameters	Training content	Communication outcome
Baseline	No device-based training	Assessment of eye opening, ocular responses.	Limited to blinking-based yes/no responses.
Initial 7 days training	1 session per day and 10 min per session	Gaze fixation training with calibration.	Simple gaze-based selection with his own name.
1–2 Months later	2–3 sessions/day, 10–30 min/session.	Training of selection of predefined words or icons, yes/no responses, and family names.	Improved ability to select predefined words or icons to express simple needs and family names.
3rd Month and later	>5 sessions/day, 30–60 min/session.	Training in phrase selection, simple typing, and WeChat-based text communication under supervision.	Generate short text messages and perform simple WeChat-based communication.

The primary goal of the training program was to improve eye scanning and gaze control as a method of transmitting eye movement signals, effectively replacing traditional keyboard and mouse interfaces. This intervention aimed to improve the patient’s quality of life and communication efficiency, facilitating tasks such as web browsing, interacting on social platforms, typing, and note-taking. Simultaneously, a multidisciplinary comprehensive rehabilitation approach was implemented to address limb movement disorders, aphasia, dysphagia, etc. The auxiliary rehabilitation devices used included PNF training of the eye muscles, passive range of motion training of the extremities, pulmonary rehabilitation therapy, an electric standing bed, isokinetic muscle strength training of the lower limbs, medium-frequency pulse electrical therapy of the extremities, acupuncture, cognitive and speech training, and swallowing therapy.

During the initial 2 months of rehabilitation training, under the supervision of a team of rehabilitation physicians and nurses, the patient received eye tracker training sessions 2–3 times daily, each lasting 10 to 30 min. Initially, he could output the names of his daughter and himself through the eye tracker. As his cognitive recovery advanced, he successfully adapted to using the eye tracker as his primary alternative communication method. Consequently, in the third month of training, the number of daily sessions increased to more than 5, with durations ranging from 30 min to 1 h. After the rehabilitative period of 3 months, the patient’s psychological adaptation and social interaction have been greatly improved, and he can achieve smooth online communication with others via WeChat. Consultations with psychological counselors indicated that the patient’s enhanced communication skills alleviated the mental strain on family members and improved the patient’s overall sense of well-being and determination to recover.

The patient showed good adherence and tolerability during eye-tracker-assisted communication training. The frequency and duration of each session were adjusted according to his attention level and fatigue. No obvious related adverse events or complications requiring discontinuation were reported by the rehabilitation team or families. After 3 months of rehabilitation, the patient continued to use the eye tracker as his primary alternative communication method in daily interaction. Because of severe motor impairment, the patient’s perspective was obtained mainly through eye-tracker-assisted communication and family interpretation. Through the device, he was able to express basic needs and emotions more effectively. Family members reported that improved communication reduced communication-related stress during daily care and enhanced the patient’s willingness to participate in rehabilitation.

**Table 2:** Reported cases of locked-in state with rehabilitative assistance by eye tracker.

Year	Author	Cases	Mean age/Gender (M/F)	Underlying conditions	Eye tracking form	Outcome
2009	Trojano et al. (2009)	1	27/M	Traumatic brain injury	Eye-tracker	Improved interaction with the environment
2013	Caligari et al. (2013)	35	NA	Amyotrophic lateral sclerosis	Eye tracking communication devices	An increase in communicative abilities and quality of life
2017	Chang et al. (2017)	3	59/(2/1)	Amyotrophic lateral sclerosis	Electro-oculogram based eye-computer interface	Successfully constructed an eye-writing system as a communication tool for patients
2018	Kim et al. (2018)	2	51/(1/1)	Amyotrophic lateral sclerosis	Electrooculogram-based human-computer interface paradigm	A new system that does not require voluntary eye movement for binary yes/no communication by patients

## 3 Discussion

This case described a patient with LIS who was treated with multidisciplinary comprehensive rehabilitation therapy and with eye-tracker as an alternative communication method. The patient had preserved consciousness and residual ocular motor responses but severe quadriplegia, anarthria, dysphagia, and communication impairment. After gradual training, the patient progressed from simple gaze-based target selection to short text generation and WeChat-based communication. This case suggests that eye-tracking technology may be a practical augmentative and alternative communication approach for selected post-stroke patients with LIS when integrated into a multidisciplinary rehabilitation program.

Long-term rehabilitation goals for patients with LIS are multiple and complex. Studies have shown that persistent impairment of motor function and communication in individuals with LIS would significantly decrease the quality of life (Rousseau et al., 2015). Therefore, the rehabilitation of communication impairment in LIS patients has gradually become a focal point for clinical doctors and patients’ families. It should be noted that multidisciplinary comprehensive rehabilitation therapy is a key part of the rehabilitation of LIS, which consists of professionals such as physiotherapists, neurologists, neuropsychologists, physical therapists, cognitive therapists, speech therapists, occupational therapists, and nurses (Surdyke et al., 2017). Casanova et al. suggested that early intervention in rehabilitation (starting on average 1 month after onset) could enhance functional recovery and reduce mortality in patients with LIS (Casanova et al., 2003). Current strategies involve the development of multi-sensory enhancement systems, leveraging social media and the Internet to replace traditional physical and voice communication methods, and establishing mobile and interconnected communication (Trojano et al., 2010). Some emerging physical rehabilitation methods have been widely studied and tested, such as eye movement ultrasound tracking systems (Kopsky et al., 2014), intracortical BCI (Milekovic et al., 2018), eye tracker (Linse et al., 2017), etc. But the advantages of eye trackers are obvious; they can visualize visual information by tracking and measuring eye position and movement information (Linse et al., 2017).

In this case, the Tobii PCEye device was associated with progressive improvement in the patient’s communication ability across three domains: basic gaze control for target selection, purposeful language output for expressing names and simple needs, and functional social communication through simple text-based interaction with caregivers, family members, and others via WeChat. Eye-tracking technology is becoming more and more popular due to its advantages of precision, low cost, portability, and user-friendliness. It can visualize visual information by tracking and measuring eye position and movement information, where eye movement reflects the processing patterns of visual information by the brain, and is widely used in survey questionnaires of paralyzed patients (Linse et al., 2017; Svernling et al., 2019). Some eye movement indicators are measured by eye tracking systems to reflect attention fixation and stimulation time (Gibaldi et al., 2017) and are collected to reflect complex cognitive information. A brief literature review supported eye trackers as a commonly used augmentative and alternative communication (AAC) approach and rehabilitation measurement for patients within locked-in state (Table 2). PubMed and Web of Science were searched using combinations of the following terms: “locked-in syndrome,” “locked-in state,” “eye tracking,” “eye tracker,” “augmentative and alternative communication,” and “electrooculography.”

The application of eye-tracker in individuals suffering from locked-in states, such as Locked-In Syndrome (LIS) and Amyotrophic Lateral Sclerosis (ALS) (Chaudhary et al., 2022; Tonin et al., 2020), presents a promising avenue for communication assessment. Caligari et al. emphasized the impact of eye-tracking communication devices on enhancing the quality of life for ALS patients, underscoring its relevance in mitigating disability (Caligari et al., 2013). Some advanced eye tracking systems appeared recently, which were combined with electrooculogram-based interfaces, offering innovative means for communication through involuntary eye movements (Chang et al., 2017; Kim et al., 2018).

Additionally, an eye tracker has emerged as a valuable tool for assessing cognitive functions in patients with ALS, which can evaluate cognitive functions in ALS patients (Kopsky et al., 2014). Eye movement parameters, including fixation duration, gaze distribution, visual scanning patterns, target selection accuracy, and response latency, may indirectly reflect attention and language processing. Based on this technology, some EEG and EOG signal datasets for cognitive evaluation have been provided (Schnakers et al., 2008; Jaramillo-Gonzalez et al., 2021). The integration of eye-tracking technology not only facilitates communication but also offers avenues for cognitive rehabilitation in severely impaired individuals. Trojano et al. demonstrate successful cognitive rehabilitation utilizing eye-based communication in a severely brain-injured patient with tetraplegia and anarthria, emphasizing its therapeutic potential (Trojano et al., 2009). This provided promising fields on rehabilitative value for eye tracking training on advanced locked-in patients.

In the present case, the eye tracker was integrated into a multidisciplinary rehabilitation program. Rehabilitation physicians, therapists, nurses, psychological counselors, and family caregivers participated in respiratory care, swallowing therapy, motor maintenance, cognitive and speech training, psychological support, nursing care, and communication practice. This integrated approach enabled the patient to express needs, interact with caregivers, and participate more actively in long-term rehabilitation.

In addition to its important applications in patients with locked-in state, the eye tracker also has important applications in the diagnosis and rehabilitation of various diseases, such as autism spectrum disorders (Mori et al., 2023), Alzheimer’s disease (Suzuki et al., 2018), Parkinson’s disease (Ba et al., 2022), and so on, accentuating its potential value beyond communication. However, there are some limitations to this technology in rehabilitation. For one thing, highly advanced locked-in patients who have completely lost autonomous eye movement may require tools based on brain-computer interface control systems rather than eye-tracking devices; for another, it is difficult to operate under a series of changing lighting and posture conditions, making it difficult to determine the optimal dwell time and required technical support.

Several limitations should be acknowledged. First, this is a single case report, and the findings cannot be generalized to all patients with LIS. Second, standardized quantitative communication measures, such as gaze-selection accuracy and typing speed, were not systematically recorded during rehabilitation because of the technical limitations of routine bedside training. Therefore, communication improvement was mainly evaluated using clinically available functional outcomes, including training progression, gaze-based target selection, WeChat-based communication, and family feedback. Third, eye-tracker-assisted communication is suitable only for patients with preserved ocular motor control, adequate attention, and sufficient visual function. Patients with complete loss of voluntary eye movement may require other assistive technologies, such as electrooculography-based systems or brain-computer interfaces.

## 4 Conclusion

This case suggests that eye-tracker-assisted communication may be an alternative communication strategy for patients with post-stroke locked-in syndrome who retain voluntary eye movements. Integrating this technology into multidisciplinary rehabilitation may support functional communication, psychosocial adaptation, and long-term recovery. Further studies with standardized outcome measures are needed.

## Data Availability

The data supporting the findings of this case report are available from the corresponding author upon reasonable request.
